# Design Optimization and Mechanical Properties of SiC Particle Reinforced Ti-Based Metallic Glass Matrix Composite

**DOI:** 10.3390/ma16155323

**Published:** 2023-07-28

**Authors:** Huawei Liu, Jing Li, Jingyao Zhang, Pan Gong, Wei Yang, Lichen Zhao, Xin Wang

**Affiliations:** 1 Hebei Key Laboratory of New Functional Materials, School of Material Science and Engineering, Hebei University of Technology, No. 5340, Xiping Road, Beichen District, Tianjin 300401, China; 2 State Key Laboratory of Materials Processing and Die & Mould Technology, School of Materials Science and Engineering, Huazhong University of Science and Technology, Wuhan 430074, China

**Keywords:** metallic glass matrix composite, titanium, silicon carbide, finite element simulation, mechanical properties

## Abstract

Ti-based bulk metallic glass (BMG) alloys have attracted widespread attention due to their strong glass forming ability, high specific strength, and good corrosion resistance. However, the poor plasticity of BMGs limits their further application in the aerospace and aircraft fields, as well as others. We optimized the composition of SiC-reinforced, Ti-based metallic glass matrix composites (MGMCs) through finite element modeling (FEM). FEM of MGMCs containing irregularly shaped SiC particles with different contents was conducted. Stress and strain analyses were conducted to evaluate the effect of the particle volume fraction on the mechanical behavior of MGMCs, and an optimization value of 30% was obtained, which is conducive to plasticity improvement. Arc melting copper mold injection casting was used to verify the optimized SiC content. The results show that the electroless nickel plating treatment effectively improves the wettability between SiC particles and the amorphous matrix, enabling the successful preparation of SiC/MGMC with a volume fraction of 29.5% through traditional injection casting. The volume fraction of SiC plays a crucial role in the transition of fracture mode from splitting to shear in MGMCs. After adding lightweight SiC particles, the yield strength, plasticity, modulus, and specific strength were improved by 25%, 1471%, 46%, and 33%, indicating that the use of nickel-plated SiC particles in MGMCs is an effective strengthening and toughening method for BMGs.

## 1. Introduction

Titanium is an abundant element in reserves on Earth, and is considered one of the most important material resources required for the development of modern technology. Benefiting from the essential properties of Ti atoms, Ti-based alloys and composites generally have high specific strength, high specific modulus, wear resistance, corrosion resistance, and good high-temperature oxidation resistance, making them widely used in many fields such as in the aerospace field, chemical engineering, biomedical materials, and in constructing ships [1,2,3]. Recently, with the development of glass-forming technology and theory, it was found that some titanium alloys can be prepared into bulk metallic glasses (BMGs) at lower cooling rates, which can further enhance their specific strength [4]. Benefiting from their disordered atomic arrangement, BMGs usually have a highly uniform chemical composition and isotropy, and lack the typical crystallographic defects such as dislocations and grain boundaries, generally resulting in a higher strength, corrosion resistance, and wear resistance than crystalline alloys with the same composition [5,6]. It should be emphasized that BMG combines two important properties, high strength, and high elasticity, with an elastic limit that is several to several tens of times that of a typical crystalline alloy, and an elastic strain of up to ~2% [7,8]. However, due to the difficulty in extending the plastic behavior that originates from the local range (limited to the shear band) to the macroscopic overall range, BMGs typically lack the ability of macroscopic room temperature plastic deformation and exhibit significant brittleness, which greatly limits their widespread application as structural materials [9,10].

### 1.1. Progress in SiC Particle Reinforced MGMCs

In order to improve the macroscopic plasticity of BMGs, one of the most effective strategies is to prepare metallic glass matrix composites (MGMCs) by introducing a crystalline second phase as a reinforcement system to promote the shear band nucleation and suppress the shear band propagation [11,12,13]. The reinforcement of MGMCs is generally designed as a crystalline second phase, including in situ-precipitated crystalline phases and ex situ-added classical reinforcements such as carbon fibers [14,15,16,17], W fibers [18,19,20,21,22], ceramic particles [23,24,25], etc. As a common reinforcing particle in crystalline matrix composites, silicon carbide (SiC) particles are also used in MGMCs through different preparation methods and metallic glass (MG) matrices.

In order to obtain the macroscopic properties of composite materials, various preparation processes have been used for the preparation of SiC-reinforced MGMCs. Li et al., used induction melting and copper mold injection casting to prepare SiC particle-reinforced, Mg-based MGMCs and found that cracks can penetrate, terminate, or bypass SiC particles when the Mg-based MGMC fractures, indicating that the presence of SiC particles is effective in preventing rapid crack propagation [26]. Lin et al., prepared SiC particle-reinforced Ti_50_Cu_28_Ni_15_Sn_7_ MGMC through mechanical alloying and hot-pressing sintering methods, and they found that partial crystallization of the glassy matrix occurred during the hot-pressing process [27]. It was also found that the addition of SiC particles improved the hardness and the fracture strength of the composite, but the sharp boundaries of the SiC particles led to brittle failure of the composite material [27,28]. Xie et al., prepared SiC particle-reinforced Ni_52.5_Nb_10_Zr_15_Ti_15_Pt_7.5_ MGMC through a spark plasma sintering (SPS) process and found that no crystallization of the matrix glass alloy occurred during the SPS process, and no interfacial reaction layer was observed [29]. They also prepared Fe-based MGMC without interfacial reaction using the SPS process and found that the addition of SiC particles is beneficial for improving high-frequency soft magnetic properties [30]. Yue et al., prepared a SiC particle-reinforced Zr_65_Al_7.5_Ni_10_Cu_17.5_ MGMC coating using laser cladding technology. It was found that an interface reaction layer containing ZrC was formed between the SiC particles and the glass phase matrix, but there was no significant negative impact on thermal stability, wear resistance, or corrosion resistance [31]. This interface reaction introduces ZrC and SiC phases into the MG matrix, which can further improve the hardness and wear resistance of the coating through the presence of a large number of ceramic phases [32]. Chen et al., prepared porous SiC-reinforced, zirconium-based MGMC using pressure infiltration and water quenching methods, and found that the SiC volume fraction had a significant impact on the deformation and fracture behavior [33,34]. When the SiC volume fraction increased to a certain extent, the compression fracture mode of the composite material changed from a shear mode to an axial splitting mode [33]. Wang et al., found that cracks in porous SiC/Ti-based MGMCs typically originate at the interface between SiC and MG matrix or within SiC particles [35]. During dynamic compression, the initiation and propagation of cracks are faster, resulting in the dynamic compressive fracture strength of the MGMC material being much lower than that of the quasi-static compression [35]. They also found that the deformation behavior under dynamic loading is more severe than static, and that the dynamic hardness of composite materials is significantly higher than their static hardness [36].

From the above document review, it can be seen that one of the challenges for MGMCs containing SiC is the control of interface reactions. Obviously, using low heating temperature processing technology (i.e., SPS) and appropriate matrix materials (i.e., Mg-based or Ni-based) is beneficial for avoiding interfacial reactions. Zhang et al., studied the wetting behavior between Zr-based alloy melt and SiC, and found that, although the wettability increased with an increasing temperature, interfacial reactions occurred at the Zr-based alloy/SiC interface [37]. They also found that the interface reaction between Zr-based MG matrix and SiC depends on the content of Zr element [38]. In our early work, we used electroless copper plating treatment on SiC particles to improve the interfacial wettability and plastic deformation ability of the composite material [39]. However, due to the large brittleness of the Mg-based MG alloy matrix, the addition of SiC particles did not cause significant macroscopic plasticity [39].

### 1.2. Application of Finite Element Analysis in Particle Reinforced MGMCs

The deformation behavior of MGMCs is limited by complex stress states, making it difficult to conduct quantitative analysis and direct experimental observation. Therefore, finite element numerical analysis based on computer simulation is widely used. Finite element modeling (FEM) can not only be used to analyze the stress and strain changes in BMG during deformation [40,41,42,43,44], but also to analyze the interfacial stress conduction in MGMCs.

The spherical second phase model was first used to analyze the stress and strain states of MGMCs under different external forces. Zhu et al., established a two-dimensional finite element model to analyze the deformation behavior of spherical Ta particle-reinforced, Zr-based MGMC. They believe that the presence of Ta particles can cause the maximum shear stress direction to deviate from the 45° plane towards the nearest particle, so the shear band can be diffused between particles [45]. Ding et al., obtained similar results in the FEA of a high-entropy, alloy-reinforced, and Zr-based MGMC [46]. They proposed that the strain hardening of particles played an important role in the bifurcation of shear bands. Ott et al., built a two-dimensional plane-strain FEM. Due to the lateral constraint imposed by the elastic matrix on particle deformation, the Ta particles did not undergo significant hardening. The nucleation of shear bands is not caused by differences in elastic properties between the matrix and particles, but by stress concentration around the particle that is induced by mismatched plastic strains [47]. They believe that, for small volume-fraction MGMCs, the presence of particles has a significant impact on the yield of MGMCs, but has almost no effect on the fracture behavior, which largely depends on the properties and behavior of the MG matrix [48]. In another FEM study of porous tungsten/Zr-based MGMC, the plastic mismatch strain of the two phases was also considered the main cause of fracture, which resulted in large stress concentration near the interface, leading to the failure of the tungsten phase or the separation of the two phases at the interface [49].

FEM analysis method has been used to analyze the influence of particle shape on the deformation behavior of MGMCs. Jiang et al., conducted FEM analysis on the deformation behavior of particle-reinforced MGMC based on a two-dimensional, eight-node plane strain element, and found that the back stress in crystal particles affects the evolution of shear bands [50]. They pointed out that the work hardening ability of particles has a significant impact on both the motion strain hardening and the strength of MGMC [50]. They also studied the influence of particle strength, shape, and volume fraction on the deformation behavior of MGMCs, and found that improving the aspect ratio and the dispersion randomness of particles would be beneficial to inhibiting the formation of main shear bands and improving the ductility of MGMCs [51,52]. Jia et al., used the FEM method to investigate the influence of different crystal geometries, including cubes/spheres/dendrites, on the deformation behavior of MGMCs [53]. They found that a complex geometric shape and favorable dendritic orientation (45°) introduce more stress concentration, thereby improving ductility. The volume fraction of the second phase should be higher than the percolation limit to block and deflect the propagation of shear bands. Bian et al., established a two-dimensional micromechanical FEM for elliptical Ta particle-reinforced MGMCs and found that, the larger the angle between the axis of charge and particle orientation, the better the toughness of MGMCs. The shape of the particles (respect ratio) has a significant impact on the plasticity improvement of MGMCs, but plays a small role in the ultimate strength [54].

To sum up, the FEM study of particle-reinforced MGMCs is mainly based on the second phase with simple shapes such as spherical, elliptical, and fibrous shapes, which do not match the actual three-dimensional morphology of SiC particles. In this work, we used linear polygonal particles as the basis for FEM analysis to study the effect of SiC volume fraction on the deformation behavior of Ti-based MGMCs, optimizing the design of an appropriate SiC volume fraction. We also used the copper mold injection casting method to prepare the optimized SiC particle-reinforced Ti-based MGMC. The mechanical properties and the deformation mechanism were investigated for validation.

## 2. Materials and Methods

### 2.1. FEM Analysis

**Figure 1:**
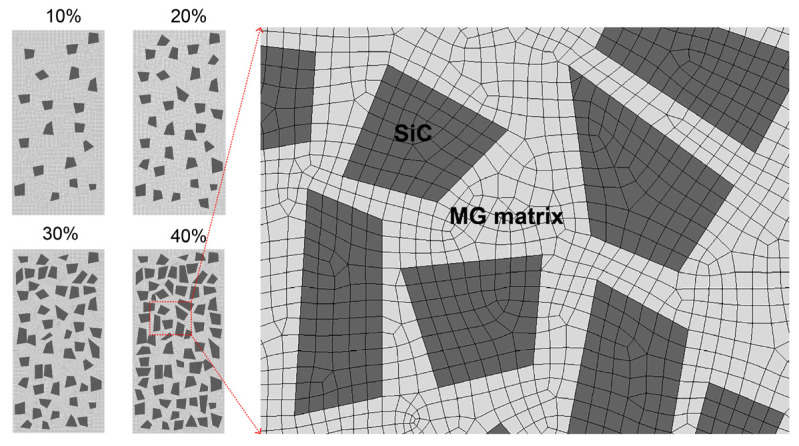
FEM models of Ti-based MGMC reinforced with different volume fractions of SiC particles, with an enlarged image showing the details of mesh division.

The typical ANSYS Workbench software was used to establish a two-dimensional finite element model, as shown in Figure 1. The necessary material parameters required for the calculation of FEM simulation are shown in Table 1. The height of the sample is 7 mm, the width is 3.5 mm, and the shape of the particles is a random linear quadrilateral with a size of approximately 0.5 mm. In order to ensure that the computer used in this work would not crash, the size of SiC particles was set to about 10 times the actual nominal size. We estimated the volume fraction of particles in the sample by calculating the ratio of total particle area to sample area, and drew two-dimensional models with volume fractions of 10%, 20%, 30%, and 40%, respectively. The grid division mode adopts automatic random allocation of four points, with a size of approximately 0.1 mm. This size is the minimum value obtained through repeated testing to ensure that the computer would not crash (as the grid size has a significant impact on computational accuracy). The upper and lower surfaces of the model were set as fixed constraints (nondeformable). To observe the stress state inside the composite material sample during the loading changes from small to large enough, a displacement of 0.2–5% was applied from top to bottom to obtain the equivalent stress cloud map and equivalent plastic strain cloud map of the sample. Here, 0.2% has no special meaning, it is a small value we randomly selected; 5% is a value selected to ensure the fracture of the composite. In addition, it should be noted that we improved the wettability of particles and titanium alloy melt by electroless nickel plating, where the nickel coating melts and disappears. Therefore, no coating was established on the surface of SiC particles in the FEM model.

### 2.2. Fabrication of MGMC

Commercial SiC particles (Guangzhou Hongwu Material Technology Co., Ltd., Guangzhou, China) with 600 mesh were used as the reinforcement of MGMC, and the particle size was approximately 10–50 μm according to the commodity specification. In order to improve the interfacial wettability, SiC particles were pretreated by common electroless nickel plating. The plating process was as follows: (1) soak in acetone for 1 h and ultrasonic cleaning for 15 min to remove organic pollutants from the surface of SiC particles; (2) soak the dried SiC particles in concentrated HF acid for 1 h to increase the surface roughness of the particles through corrosion for roughening treatment; (3) soak in HCl solution of SnCl_2_ for 30 min for sensitization, and activate the particles with HCl solution of PdCl_2_ after cleaning, the treated SiC particles must be reduced with sodium hypophosphite solution after activation; (4) carry out nickel plating treatment using nickel plating solutions prepared with NiSO_4_·6H_2_O, NaC_6_H_5_O_7_·2H_2_O, NaH_2_PO_2_·H_2_O, CH_3_COONa, NH_4_Cl, and NaOH at a plating temperature of 60 °C.

Fragments of Ti, Zr, Be, Fe, and Cu with a purity of more than 99.9% were used as raw materials. Analytical balance with an accuracy of 0.0001 g (Mettler, ME204E, Columbus, OH, USA) was used to weigh the alloy ingredient of (Ti_41_Zr_25_Be_28_Fe_6_)_98_Cu_2_ [55]. Under the protection of high-purity argon gas, the MG master alloy ingot for matrix alloy of MGMC was prepared using a vacuum arc furnace (Shanghai MTINST, DHL-600, Shanghai, China). In order to ensure the uniformity of the composition, special attention should be paid to the following aspects in the experimental operation: firstly, ensure that the vacuum degree reaches 1 × 10^−3^ Pa or above to reduce the oxygen content in the furnace body; secondly, high-purity Ar with a purity of 99.9999% should be used to avoid introducing impurity gas molecules; thirdly, it is necessary to frequently replace pure titanium ingots used for oxygen absorption to avoid weakening their oxygen absorption effect; fourthly, during the re-melting process, the ingot needs to be flipped up and down 5 times to avoid gravity segregation; fifthly, the weight of the obtained ingots must be carefully weighed, and ingots with a mass change of no more than 0.5% before and after melting are considered qualified samples for subsequent MGMC preparation (ingots exceeding 0.5% are considered unqualified samples and discarded). Then, the nickel-plated SiC particles and the (Ti_41_Zr_25_Be_28_Fe_6_)_98_Cu_2_ ingot fragments were placed in the vacuum arc furnace (Shanghai MTINST, DHL-600, Shanghai, China) to prepare the SiC/(Ti_41_Zr_25_Be_28_Fe_6_)_98_Cu_2_ composite master alloy material. Finally, a round rod-shaped specimen with a diameter of 2 mm and a length of about 70 mm was prepared using the copper mold injection casting method [60] by an injection casting furnace (Shanghai MTINST, VIMC-1000, Shanghai, China). The vacuum degree of the furnace chamber is −1.0 × 10^−2^ Pa, and the pressure of the injection-cast Ar was about 2 MPa.

### 2.3. Microstructural Characterization

An X-ray diffractometer (XRD, Bruker D8 Advance, Cu Kα radiation, Billerica, MA, USA) was used to examine the phase composition of SiC particle samples and MGMC rod samples with a scanning speed of 10°/min and a scanning angle range of 20–80°. A differential scanning calorimeter (DSC, Setaram Themys ONE, Cranbury, NJ, USA) was used to measure the thermal stability of the as-prepared samples under N_2_ protection at a heating rate of 20 K/min. A scanning electron microscope (SEM, Hitachi S-4800, Tokyo, Japan) was used to examine the microstructure of the plated SiC particles and the rod MGMC samples. An EDS detector assembled on the S-4800 SEM was used to determine the chemical composition of the matrix of the composite material.

### 2.4. Mechanical Property Testing

A Vickers micro hardness tester (SHIMADZU, HMV-2TE, Kyoto, Japan) was used to test the micro hardness of composite materials, with a loading load of 980 mN and a loading time of 15 s. Metallographic microscope (Olympus, BX41RF, Tokyo, Japan) was used to take photos of hardness indentation. A slow diamond cutting machine (Kejing, SYJ-150, Shenyang, China) was used to cut a circular rod with a diameter of 2 mm into a cylindrical sample with a length of 4 mm for compression testing. A computer-controlled electro-hydraulic servo universal testing machine (Guanteng, PA200, Changchun, China) was used to test the compressive mechanical properties of the MGMC and BMG samples with a strain rate of 0.5 mm/min. To ensure the reliability of the data, each sample was tested at least 3 times, and the mechanical property was taken from the average value of the samples. The density of different as cast samples was tested based on the Archimedes drainage method, and the specific strength was evaluated by dividing the yield strength obtained from the compression experiment by the density.

## 3. Results

### 3.1. Optimization of SiC Particle Volume Fraction

**Figure 2:**
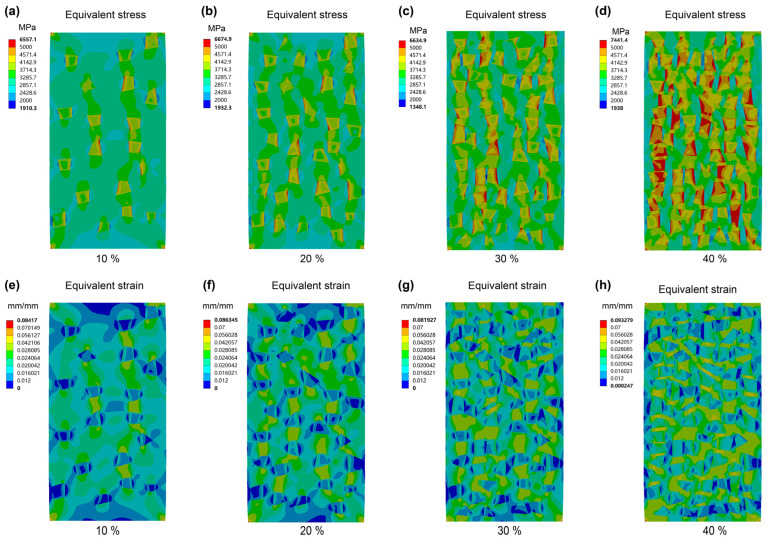
Contour maps of equivalent stress analysis at 5% deformation for MGMC samples with different SiC volume fractions of (**a**) 10%, (**b**) 20%, (**c**) 30%, and (**d**) 40%, and the corresponding equivalent plastic strain analysis of samples with different SiC content of (**e**), 10%; (**f**), 20%; (**g**), 30; and (**h**), 40%.

Figure 2 shows the FEM analysis results of the Ti-based MGMC samples reinforced with different volume fractions (*V_f_*) of SiC particles under a total deformation of 5%. From the equivalent stress analysis shown in Figure 2a–d, it can be seen that the maximum compressive stress in the sample occured within the SiC particles, and there is a gradual increasing trend with the increase in *V_f_*. For the stress distribution on the matrix, when the *V_f_* value is less than 20%, the maximum stress (green area) is limited to the area between two adjacent particles arranged vertically, indicating that yielding or fracture tends to occur along the axial direction (splitting fracture mode). When the *V_f_* value reaches over 30%, the maximum stress range exhibits transverse crosslinking, and the higher the *V_f_*, the stronger the transverse crosslinking. As a result, the MGMC sample with a *V_f_* value of 40% exhibited the most stress concentration zones in the 45° direction (maximum shear stress direction), which clearly facilitated the transition of the fracture mode from the splitting mode to the shear mode.

Figure 2e–g shows the FEM analysis results for the equivalent plastic strain, where the low strain zone is essentially located within the SiC particles. This can be attributed to the fact that the Young’s modulus of a SiC particle (~400–550 GPa [57,58,61,62]) is about six times that of MG matrix alloy (~100 GPa [56,63,64]), resulting in the smaller deformation of SiC particles under the same stress-bearing conditions. However, as *V_f_* increases, the low strain region in the SiC particles gradually decreases, which can be attributed to the increasing effect of SiC *V_f_* on the maximum stress under the same deformation amount, as shown in Figure 2a–d. In addition, when the *V_f_* value is less than 20%, the distribution of the maximum strain zone (green and yellow areas) is consistent with the equivalent stress, and also tends to be vertically distributed. When the *V_f_* value reaches over 30%, the maximum equivalent plastic strain zone exhibits multiple 45° distributions, which means that the plastic deformation zone in the sample can extend along the main shear direction to the entire sample. In other words, this stress distribution contributes to the formation of the multiple shear banding mode.

The FEM analysis results for equivalent stress and equivalent plastic strain both support that *V_f_* is a key parameter which determines the deformation fracture mode of SiC-reinforced MGMCs. From the results in Figure 2, it can be seen that the ideal SiC *V_f_* value for achieving good ductility should be above 30–40%. Considering that, if *V_f_* is too large, SiC particles are prone to agglomerate and form defects during the fabrication process, which is not conducive to injection molding, the *V_f_* should not be too large.

### 3.2. Fabrication and Microstructure of Composite Materials

**Figure 3:**
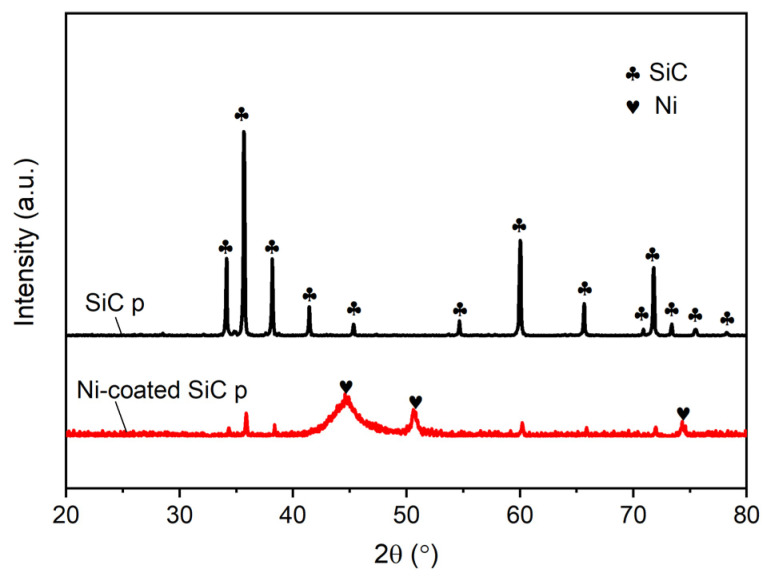
XRD patterns of SiC particles before and after electroless Ni plating.

Figure 3 shows the XRD patterns of the original SiC particles and the SiC particles after electroless Ni plating. The sharp diffraction peaks in the XRD pattern of the as-received SiC particles correspond to SiC crystals, indicating that the tested original powder sample contains a single SiC phase and its purity is high. For the XRD pattern of the Ni-coated SiC particles, in addition to the weak peaks of crystalline SiC, there is also a group of diffraction peaks that have undergone significant broadening, with peak angles consistent with those of Ni crystals. We know that the products of alkaline electroless nickel plating are P-rich, Ni-based amorphous alloy or nanocrystalline alloy, which may be the main reason for the broadening of the Ni peaks. It should be noted that the height of the Ni peak is significantly higher than the main peak of SiC, indicating that the surface of SiC particles has been successfully coated with Ni after plating. The existence of weak peaks in SiC can be attributed to the fact that the electroless Ni coating is very thin, thus, the conductive X-ray can penetrate the coating. In addition, some SiC particles were missed during electroless plating, resulting in their failure to form a surface coating and exposing a certain amount of surface to be detected.

**Figure 4:**
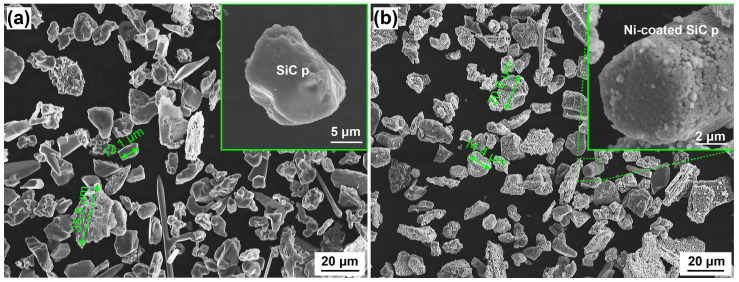
SEM images of SiC particle samples before electroless nickel plating (**a**) and after electroless nickel plating (**b**).

Figure 4 compares the microstructures of the SiC particles before and after electroless nickel plating. Figure 4a shows the microstructure of commercial SiC, indicating that the size of these particles ranges from ~10 μm–~40 μm. Most particles were in the form of single particles, with sharp edges and smooth surfaces, as shown in the inset in Figure 4a, which is not conducive to wettability between the Ti-based MG alloy melt and the SiC particles. Meanwhile, some particles showed a status of loose clusters with rough surfaces—in our opinion, such particles may result in the formation of cast defects which would induce a low strength and Young’s modulus of MGMCs due to the surface status. Therefore, it is necessary to carry out electroless nickel plating to modify the surface structure of SiC particles. Figure 4b shows the microstructure of SiC particles after electroless nickel plating. It can be seen that, after electroless nickel plating, the surface of the SiC particles is covered with a nickel coating as shown in the inset of Figure 4b. It is worth noting that, as the size of the SiC particle increases, the coverage of the Ni coating plated on the particle surface becomes better, while small particles are prone to be partially missing plating.

**Figure 5:**
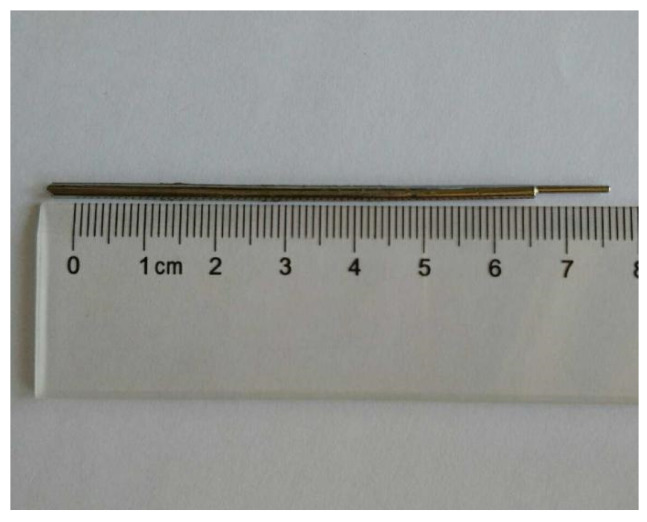
Typical macroscopic optical image of as-cast SiC/(Ti_41_Zr_25_Be_28_Fe_6_)_98_Cu_2_ MGMC rod.

Figure 5 shows an optical photo of the MGMC rod prepared by an injection casting system. The diameter of the cast sample is 2 mm and the length is 70 mm, which is consistent with the size of the inner cavity of the copper mold used, indicating that the composite material melt could completely fill the mold. We know that solid–liquid mixtures containing a large number of solid particles typically have poor flowability, which is not conducive to the formation of castings. In this work, a mixture of (Ti_41_Zr_25_Be_28_Fe_6_)_98_Cu_2_ melt and SiC particles was injected into a copper mold using high-pressure Ar gas up to 2 MPa, which relatively weakened the flowability requirements for the mixed liquid. Under high-pressure injection casting conditions, the forming process is mainly influenced by the heating temperature (which affects viscosity and fluidity) and the content of solid SiC particles. Figure 5 shows that, when the addition amount of SiC is ~30%, its flowability meets the requirements of spray casting molding. We also attempted to achieve a 50% addition of SiC, but it is impossible to obtain cast rods for mechanical performance testing due to the poor formability. Therefore, it is not advisable to add higher volume fractions of solid particles (i.e., over 50%) for injection casting. This is consistent with the limit value calculated by the granular soil model (47%) [65], indicating that the permeation behavior of metal melt between solid particles is similar to that of water in soil.

**Figure 6:**
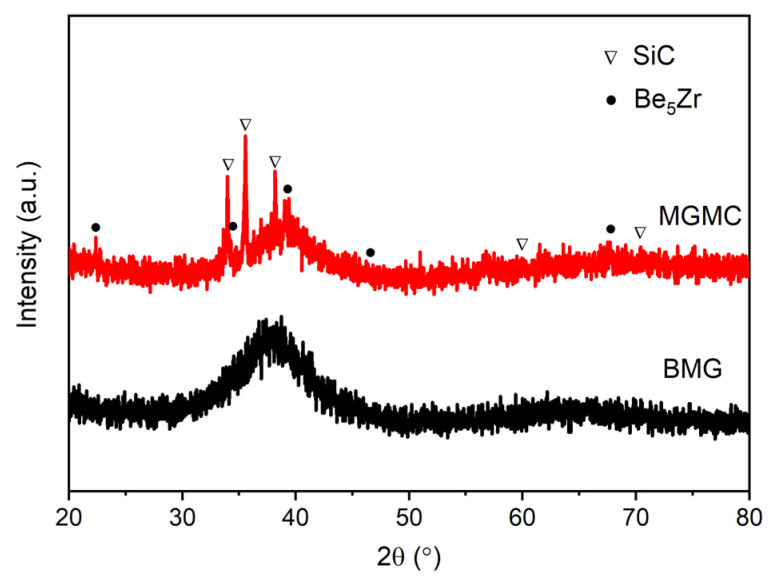
XRD patterns of SiC/(Ti_41_Zr_25_Be_28_Fe_6_)_98_Cu_2_ MGMC and (Ti_41_Zr_25_Be_28_Fe_6_)_98_Cu_2_ BMG sample.

Figure 6 shows the XRD patterns of the MGMC sample and the (Ti_41_Zr_25_Be_28_Fe_6_) _98_Cu_2_ BMG sample, comparatively. It is known that, due to the lack of long-range ordering in amorphous structures, typical XRD patterns typically exhibit diffuse peaks within a specific angle range. Obviously, both patterns in Figure 6 have this typical feature, indicating that both samples contained a certain amount of amorphous phase. For the MGMC sample, in addition to the diffuse peaks belonging to the amorphous phase, there are also some sharp diffraction peaks corresponding to the crystalline Be_5_Zr and SiC, respectively. This shows that, after adding Ni-plated SiC particles, a certain amount of crystalline phase precipitates onto the amorphous matrix. It is interesting that the precipitated crystalline phase is not an intermediate phase containing Si and C, and this indicates that SiC did not undergo significant decomposition during the preparation process. Here, the formation of crystalline phases may be related to the change in matrix composition caused by the introduction of Ni, C, and Si atoms, leading to a decrease in the glass forming ability (GFA) of the matrix alloy and resulting part crystallization. In addition, by comparing the XRD patterns of the MGMC sample in Figure 6, it can be seen that the height of the main diffraction peaks of SiC is significantly higher than that of Be_5_Zr, indicating that the amount of SiC is much higher and that SiC is the main second phase in the MGMC sample.

**Figure 7:**
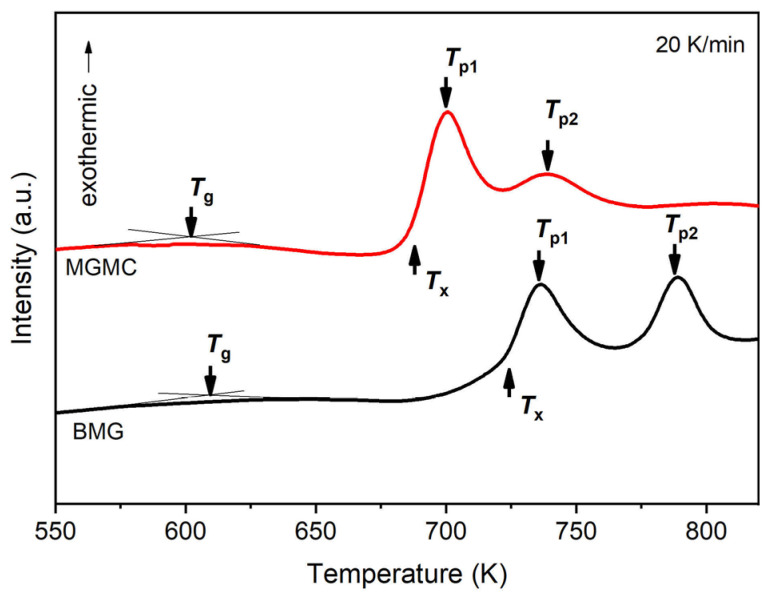
DSC curves of SiC/(Ti_41_Zr_25_Be_28_Fe_6_)_98_Cu_2_ MGMC and (Ti_41_Zr_25_Be_28_Fe_6_)_98_Cu_2_ BMG samples.

To further confirm the presence of amorphous phases, the DSC curves of the MGMC sample and the BMG sample were tested, as shown in Figure 7. For BMG, a glass transition occurs when it is heated to approximately 609 K, and crystallization begins at 724 K, forming a clear exothermic peak with the peak temperature *T_p1_* being 735.7 K. The first crystallization is followed by a second crystallization, forming a second exothermic crystallization peak, with a peak temperature *T_p2_* of 788.9 K. This is a typical behavior of amorphous alloys during heating, which is consistent with the results in the literature [55]. For the MGMC sample, the DSC curve exhibited similar heat absorption and release behavior to the BMG sample, but the glass transition temperature *T_g_*, the onset crystallization temperature *T_x_*, the first crystallization peak temperature *T_p1_*, and the second crystallization peak temperature *T_p2_* were all lower than that of the BMG sample (as shown in Table 2). It was demonstrated that the MGMC samples contain amorphous phases, but their thermal stability was reduced compared to the BMG samples. In addition, the difference between *T_g_* and *T_x_* is referred to as the width of the supercooled liquid phase zone *ΔT* (*T_g_* − *T_x_*), which can also be regarded as an indicator of the thermal stability of the sample. The *ΔT* value of the MGMC sample was decreased by up to 30 K compared to the BMG sample, which also proves that its thermal stability was deteriorated. This can be attributed to the presence of SiC particles in the MGMC sample, which leads to the preferential nucleation of crystalline precipitates on the SiC surface, requiring lower energy and resulting in the formation of a lower *T_x_*.

**Figure 8:**
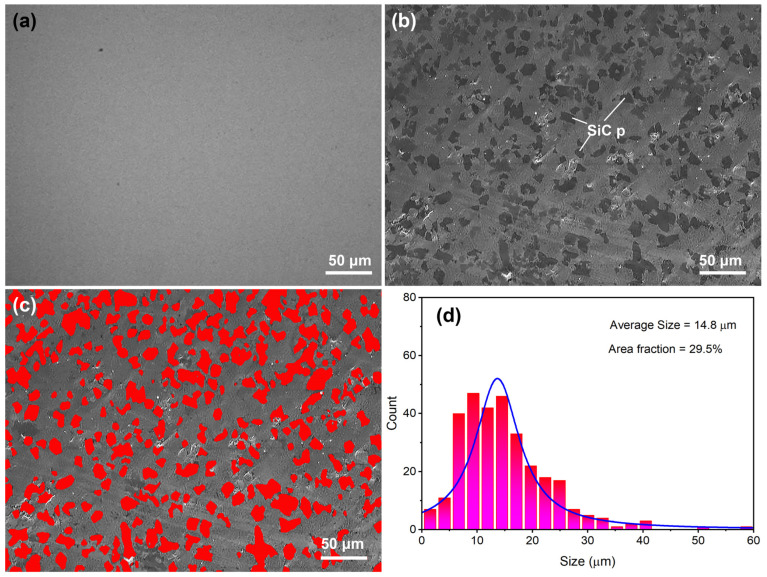
Typical SEM images of (**a**) (Ti_41_Zr_25_Be_28_Fe_6_)_98_Cu_2_ BMG and (**b**) SiC/(Ti_41_Zr_25_Be_28_Fe_6_)_98_Cu_2_ MGMC, (**c**) partial rendering image of (**b**), and (**d**) histogram of particle size distribution and area fraction measurement results.

Figure 8 shows typical SEM images of (Ti_41_Zr_25_Be_28_Fe_6_)_98_Cu_2_ BMG and SiC/(Ti_41_Zr_25_Be_28_Fe_6_)_98_Cu_2_ MGMC. Figure 8a shows the microstructure of the BMG sample, which clearly shows that there are no other phases present on the gray matrix, indicating that the BMG sample prepared in this work had a full amorphous structure. This is consistent with the results in Figure 6 and Figure 7. Figure 8b shows the SEM image of the MGMC sample. There are many dark gray irregular particles evenly distributed on the light gray matrix. From the particle morphology, it can be inferred that these particles are SiC particles. To analyze the content of the particles, we stained dark gray particles (Figure 8c) and analyzed the size distribution of the particles using Image Pro Plus 6.0 software, as shown in Figure 8d. The average size of the particles was 14.8 μm and the particle area fraction was 29.5%, indicating that the prepared MGMC sample reached the preset SiC volume fraction value (30%) according to the FEM analysis results.

**Figure 9:**
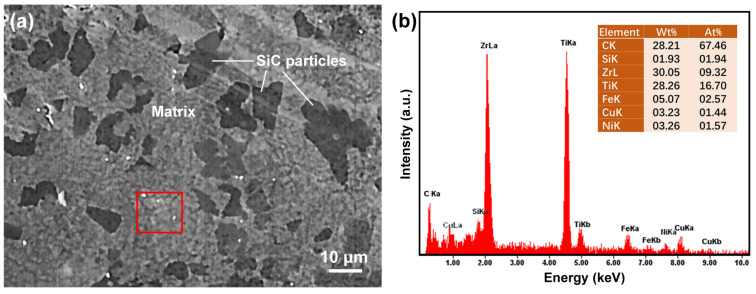
(**a**) High magnification SEM image of SiC/(Ti_41_Zr_25_Be_28_Fe_6_)_98_Cu_2_ MGMC and (**b**) EDS analysis results of the matrix area marked by the red box in (**a**).

Figure 9a shows a high magnification SEM image of as-cast SiC/(Ti_41_Zr_25_Be_28_Fe_6_)_98_Cu_2_ MGMC. The dark gray irregularly shaped particles are the SiC phase, and there is no obvious phenomenon of Ni coating on the surface of the SiC particles, indicating that the electroless Ni coating melted and might have entered the matrix during the preparation process of the MGMC. The continuous area between the dark gray SiC particles is the matrix of the MGMC. Under high magnification SEM, the matrix exhibited a pitted shape formed by mixing small particles of one darker color and another lighter color, indicating the presence of multiple phases in the matrix. Based on the XRD analysis results of the MGMC sample in Figure 6, we believe that the matrix phase should actually consist of two phases: the metallic glass phase and the Be_5_Zr crystalline phase. In order to analyze the whereabouts of the Ni layer, the chemical composition of the matrix was determined by EDS, and the results are shown in Figure 9b. Since EDS cannot measure elements with an atomic number less than 12, the values for the contents of C and Be are inaccurate. At the same time, the presence of SiC particles below the sample testing area may have also caused some interference in the testing. Therefore, the composition analysis based on EDS should exclude the contents of C, Si, and Be elements. In this way, the content ratio of the remaining elements in the nominal composition is Ti:Zr:Fe:Cu = 55.4:33.8:8.1:2.8. For the MGMC samples, the proportion of other elements after excluding the interfering elements is approximately Ti:Zr:Fe:Cu:Ni = 52.8:29.5:8.1:4.6:5.0. It can be confirmed that Ni atoms in the SiC surface coating entered the matrix, and due to the introduction of Ni, the relative content of Ti and Zr in the matrix decreased while the Cu content increased, ultimately resulting in the matrix composition deviating from the nominal composition. This will alter the GFA and thermal stability of the matrix, which is consistent with the XRD and DSC test results shown in Figure 6 and Figure 7.

### 3.3. Mechanical Properties

**Figure 10:**
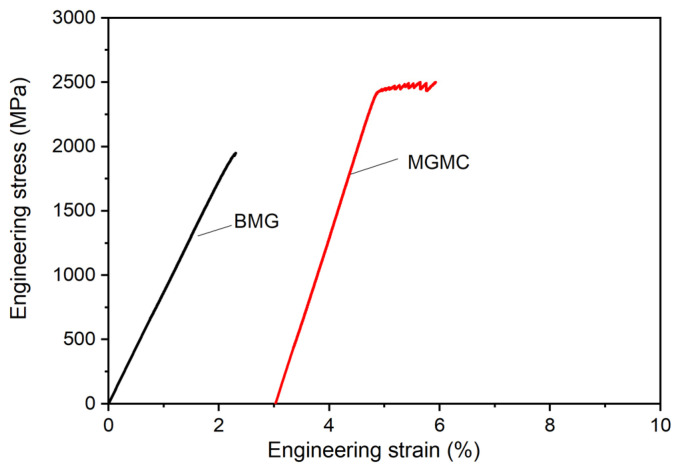
Compression engineering stress–strain curves of BMG and MGMC.

Figure 10 shows the quasi-static compression stress–strain curve of Ti-based BMG alloy and SiC/MGMC, and Table 3 shows the calculation results of the conditional yield strength *σ_0.2_*, fracture strength *σ_f_*, elastic strain *ε_e_*, plastic strain *ε_p_*, elastic modulus *E*, and specific strength *σ_sp_* measured from the stress–strain curves. The compressive fracture strength *σ_f_* of the BMG sample is 1955 MPa, and the plastic strain *ε_p_* is almost “0,” indicating that its mechanical behavior exhibits brittle characteristics. The compressive fracture strength *σ_f_* of the MGMC sample after adding SiC is 2453 MPa, and the plastic strain *ε_p_* is 1.1%, indicating that the compressive strength of the MGMC was enhanced and the plastic deformation ability was significantly improved after adding Ni-plated SiC particles. It is worth noting that the slope of the elastic deformation stage on the stress–strain curve of MGMC is larger than that of BMG sample, indicating a significant increase in its elastic modulus *E*. This is because the *E* value of SiC particle is higher than that of MG matrix, which increases the modulus of the composite material. In addition, the decrease in density and increase in strength of the MGMC resulted in a further increase in its specific strength.

**Figure 11:**
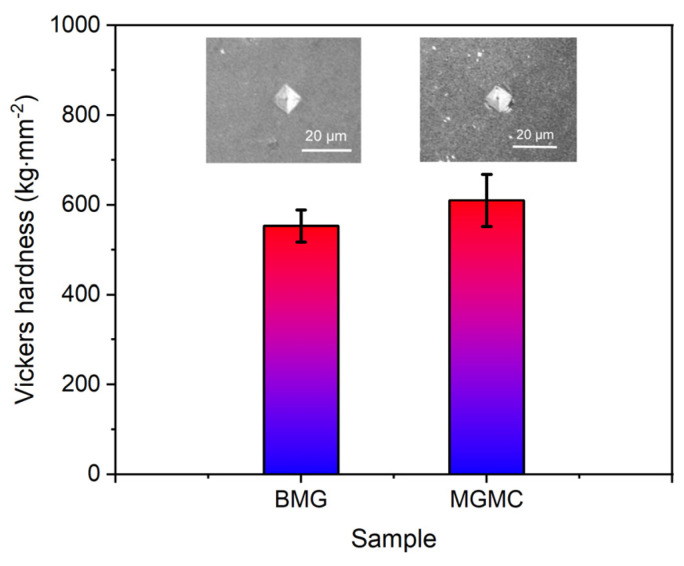
Vickers hardness analysis of the BMG and MGMC sample with typical optical photos of indentations.

Figure 11 shows the results of Vickers microhardness analysis for BMG and MGMC samples. The hardness of the BMG sample was 553 ± 36 kg·cm^−2^, and the hardness of the MGMC sample was 612 ± 58 kg·cm^−2^. It can be seen that the hardness of the MGMC sample was significantly higher than that of the BMG sample, which is consistent with the yield strength results listed in Table 3. The increase in the average hardness of the MGMC can be attributed to the introduction of high hardness-enhancing phase SiC. In addition, typical optical photos of hardness indentations on the two samples are also shown in comparison in Figure 11. The indentation on the BMG sample has a typical diamond shape, and the boundary of the indentation is very clear. The indentation of the MGMC sample is slightly flat, and there are circular stripes stacked around it, which may be due to the presence of SiC particles around the indentation, affecting the indentation behavior of the hardness tester’s indenter. In addition, the hardness indentation pattern of circular shear band stacking is a typical feature of ductile BMG, indicating that the toughness of the MGMC samples was better.

## 4. Discussion

The toughening mechanism of BMGs using the MGMC strategy is mainly related to the properties of the second phase [13]. Numerous studies have shown that adding plastic metallic particles is beneficial for toughening, as plastic particles can undergo plastic deformation earlier than the MG matrix, as the applied load increases, and absorb the deformation energy of the sample through the deformation of the particles themselves [66,67,68,69,70]. However, the strength of the plastic phase is generally low, leading to an improvement in MGMC plasticity accompanied by an obvious strength degradation, inevitably weakening the MGMCs competitive advantage of high strength. The use of high-strength ceramic particles to prepare MGMC has not achieved significant toughening effects in many research cases [26,27,28,29,30,31,32,33,34,35,36]. Most scholars attribute this to the poor plastic deformation capability of high hard second-phase particles [25,26,71], which easily induce crack initiation and form catastrophic fractures in series with each other. However, our calculation results indicate that, when the *V_f_* value of SiC particles increases to over 30%, internal stress conditions that are more favorable for shear deformation can be obtained under large deformation conditions.

**Figure 12:**
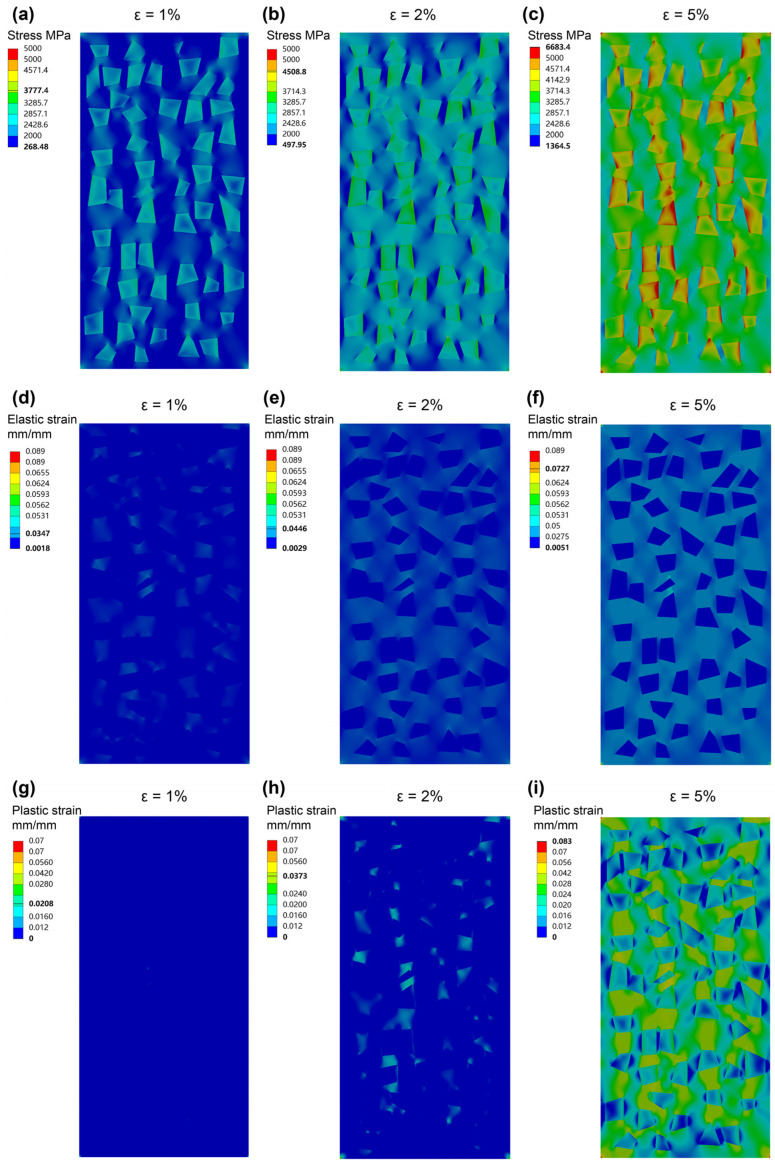
The Contour maps of equivalent stress (**a**–**c**), equivalent elastic strain (**d**–**f**), and equivalent plastic strain (**g**–**i**) calculated by FEM based on the 30%-SiC-MGMC samples under different strains of 1%, 2%, and 5%, respectively.

Figure 12 shows the Contour plots of the equivalent stress, equivalent elastic strain, and equivalent plastic strain of the 30%-SiC-MGMC samples under different deformation amounts. Obviously, under the condition of 2% strain, SiC particles in MGMC can induce cross stress distribution, which is beneficial for the uniform loading of different parts of the sample. SiC particles have an irregular shape, which may result in significant stress concentration at local edges and corners, which, in turn, may lead to local failure. However, our calculation results indicate that, due to the large elastic deformation of the MG matrix, this very limited stress concentration phenomenon is not as large as expected when the deformation is less than 2%. Figure 12b shows that the stress on the matrix is mainly distributed between particles. In two adjacent particles arranged vertically, this phenomenon is especially obvious. It is already known that the maximum stress distribution line between circular particles is very straight. In contrast, the regularity of the maximum stress distribution line between irregularly shaped particles is worse, which is beneficial for the random deflection of shear bands. The stress near circular particles is mainly concentrated in four directions: up, down, left, and right, while the stress concentration points of irregular particles are more random. When the number of particles is large, this randomness is beneficial for dispersing stress and avoiding the maximum stress overlapping with the main shear direction.

From the distribution of the equivalent plastic strain (Figure 12g–i), when the deformation amount is about 2%, plastic deformation occurs almost everywhere in the MGMC sample, which means that the nucleation sites of shear bands are more numerous and dispersed, which is conducive to the formation of multiple shear bands. However, this requires the matrix to have sufficient intrinsic toughness, which means that the matrix should have sufficient resistance to early local shear band nucleation before the formation of multiple shear bands. Therefore, a matrix with poorer intrinsic toughness (i.e., Mg-based MG [39]) often undergoes catastrophic fracturing before SiC plays a role in deflecting shear bands.

In addition to the *V_f_* of SiC, mentioned earlier, other factors such as casting defects and crystalline precipitates in the matrix/interface can also affect the mechanical properties of MGMC. In this work, the mechanical properties, such as Young’s modulus *E*, yield strength *σ_0.2_*, and fracture strength of composite materials *σ_f_*, do not meet the composite law describing the relationship between the volume fraction and performance of reinforcements in the field of composite materials. For example, the yield strength *σ_C_* of the MGMC should follow *σ_C_* = *σ_M_*·(1 − *V_f_*) + *σ_SiC_*·*V_f_*, where *σ_M_* and *σ_SiC_* refer to the yield strength of the matrix alloy and SiC particle, respectively. However, the test value 2453 MPa is lower than the calculated ones 5800–7300 MPa (theoretical strength value of polycrystalline SiC, 15–20 GPa [58]). This can be attributed to the following aspects: firstly, the casting defect caused by the high content of SiC particles in the composite, including pores, insufficient infiltration, inclusions, etc., will cause the decline of the nominal strength of the MGMC due to the inability to share the external load (weakening the strengthening effect); secondly, the possible interface reactions at the interface may result in unfavorable interface segregation, including the formation of small amounts of carbides, silicide, etc., through the local decomposition of SiC, which will cause interface cracking through stress concentration; thirdly, the dissolution of the electroless nickel plating layer leads to changes in the composition of the matrix, deviating from the optimal GFA range and causing the precipitation of certain nanoscale crystalline phases (Figure 6), which will increase the brittleness of the MGMC matrix.

In order to improve the strengthening effect of SiC particles on MGMC, future work should focus on modifying the interface microstructure of composite materials. In short, the interface reaction on the SiC surface can be reduced through appropriate matrix alloy selection, optimization of the composite material preparation process, and interface structure regulation technology. Our present work has confirmed that the electroless nickel plating treatment on the surface of SiC particles can improve the wettability between the matrix alloy and SiC particles, which also shows the potential for regulating the interface microstructure of MGMC. In the future, the structure and composition of the electroless Ni coating should be optimized to reduce the drastic changes in the matrix composition caused by the melting of the Ni layer. For example, the content of P and O in the coating can be reduced by changing the pH value and the composition of the plating solution. The addition of refining agents can also refine Ni grains to improve the coverage rate of the coating on the SiC particles.

## 5. Conclusions

This article uses the FEM method to study the micro mechanical behavior of SiC particle-reinforced, Ti-based MGMC, and obtains the optimal volume fraction based on the calculation results. Subsequently, 30%-volume-fraction, nickel-plated SiC/(Ti_41_Zr_25_Be_28_Fe_6_)_98_Cu_2_ MGMC was prepared using an arc melting copper mold spray casting method, and its mechanical properties were tested. The main conclusions drawn are as follows:

(1) The FEM analysis based on irregular SiC particles shows that, when the volume fraction is below 20%, the maximum stress and strain tend to be distributed parallel to the force axis, which may lead to splitting fracture; when the volume fraction is higher than 30%, the maximum stress and strain tend to form a cross network, which is conducive to shear fracture;
(2) Electroless nickel plating on the surface of SiC particles can significantly improve the interfacial wettability of composite materials. As a result, MGMC with a SiC volume fraction of 29.5% was successfully prepared using copper mold injection casting. The Ni-coated SiC particles exhibited dispersed distribution in the amorphous matrix, but resulted in the precipitation of a small amount of Be_5_Zr phase in the amorphous matrix, indicating the GFA degradation of the matrix alloy and the occurrence of partial crystallization;
(3) The glass transition temperature and the crystallization temperature of the MGMC sample significantly decreased, indicating a reduction in the thermal stability of the composite. This can be attributed to the presence of Ni-coated SiC, which changes the composition of the MG matrix, resulting in reduced thermal properties of the matrix. In addition, the presence of SiC particles reduces the free energy barrier of crystalline-phase precipitation due to heterogeneous nucleation, thereby lowering the crystallization temperature;
(4) The compression test showed that the compressive strength and hardness of the prepared MGMC were significantly increased compared to the matrix MG alloy, reaching over 2500 MPa and 612 kg·cm^−2^, and the plastic strain prior to fracture increased from almost “0” to about 1.1%. The use of lightweight reinforcements also resulted in a high specific strength of 4.8 × 10^5^ Nm/kg;
(5) The introduction of electroless Ni-plated SiC particles play multiple roles in weight lightening, strengthening, and toughening, providing a basis for the design and development of MGMCs. This work sheds light on the manufacturing strategy of high specific strength and wear-resistant small parts needed in the fields of consumer electronics, unmanned aerial vehicles, and robots.

## Figures and Tables

**Table 1:** Property data of the matrix and SiC reinforcement phase used for designing MGMC during ANASYS simulation.

Materials	Properties		Reference
MG matrix	density	5.36 g·cm^−3^	[55]
	Compression strength	1955 MPa	This work
	Young’s modulus	100 GPa	[56]
	Poisson’s ratio	0.35	[56]
SiC particle	density	3.2 g·cm^−3^	[57]
	Compression strength	15 GPa	[58]
	Young’s modulus	500 GPa	[57]
	Poisson’s ratio	0.2	[59]

**Table 2:** Thermal properties measured from the DSC curves of SiC/(Ti_41_Zr_25_Be_28_Fe_6_)_98_Cu_2_ MGMC and (Ti_41_Zr_25_Be_28_Fe_6_)_98_Cu_2_ BMG samples.

Sample	*T_g_* (K)	*T_x_* (K)	*T_p1_* (K)	*T_p2_* (K)	*ΔT* (K)
BMG	609.1	724.2	735.7	788.9	115.1
MGMC	601.7	687.3	700.1	739.1	85.6

**Table 3:** Mechanical properties of BMG and MGMC samples measured from compression engineering stress–strain curves.

Sample	*ρ* (g/cm^3^)	*σ*_0.2_ (MPa)	*σ_f_* (MPa)	*ε_e_* (%)	*ε_p_* (%)	*E* (GPa)	*σ_sp_* (Nm/Kg)
BMG	5.36	1955	1955	2.1	0.07	93	3.6 × 10^5^
MGMC	5.04	2453	2502	1.8	1.1	136	4.8 × 10^5^
Improvement rate	−6%	25%	28%	−14%	1471%	46%	33%

## Data Availability

Any further detailed data may be obtained from the authors upon a reasonable request.
